# Chromosomal evolution and phylogeny in the Nullicauda group (Chiroptera, Phyllostomidae): evidence from multidirectional chromosome painting

**DOI:** 10.1186/s12862-018-1176-3

**Published:** 2018-04-25

**Authors:** Anderson José Baia Gomes, Cleusa Yoshiko Nagamachi, Luis Reginaldo Ribeiro Rodrigues, Malcolm Andrew Ferguson-Smith, Fengtang Yang, Patricia Caroline Mary O’Brien, Julio Cesar Pieczarka

**Affiliations:** 1 Laboratório de Citogenética, CEABIO, ICB, Universidade Federal do Pará, Av. Bernardo Sayão, sn. Guamá, Belém, Pará 66075-900 Brazil; 2 Laboratório de Genética e Biodiversidade, ICED, Universidade Federal do Oeste do Pará, Belém, Brazil; 3 Instituto Federal do Pará, Abaetetuba, Pará Brazil; 4 CNPQ Researcher, Brasilia, Brazil; 5 Department of Veterinary Medicine, Cambridge Resource Centre for Comparative Genomics, University of Cambridge, Cambridge, UK; 6 Cytogenetics Facility, Welcome Trust Sanger Institute, Hinxton, UK

**Keywords:** Chromosome phylogeny, Molecular cytogenetics, Bat evolution, Genome mapping

## Abstract

**Background:**

The family Phyllostomidae (Chiroptera) shows wide morphological, molecular and cytogenetic variation; many disagreements regarding its phylogeny and taxonomy remains to be resolved. In this study, we use chromosome painting with whole chromosome probes from the Phyllostomidae *Phyllostomus hastatus* and *Carollia brevicauda* to determine the rearrangements among several genera of the Nullicauda group (subfamilies Gliphonycterinae, Carolliinae, Rhinophyllinae and Stenodermatinae).

**Results:**

These data, when compared with previously published chromosome homology maps, allow the construction of a phylogeny comparable to those previously obtained by morphological and molecular analysis. Our phylogeny is largely in agreement with that proposed with molecular data, both on relationships between the subfamilies and among genera; it confirms, for instance, that *Carollia* and *Rhinophylla*, previously considered as part of the same subfamily are, in fact, distant genera.

**Conclusions:**

The occurrence of the karyotype considered ancestral for this family in several different branches suggests that the diversification of Phyllostomidae into many subfamilies has occurred in a short period of time. Finally, the comparison with published maps using human whole chromosome probes allows us to track some syntenic associations prior to the emergence of this family.

**Electronic supplementary material:**

The online version of this article (10.1186/s12862-018-1176-3) contains supplementary material, which is available to authorized users.

## Background

The family Phyllostomidae is the third most speciose within the order Chiroptera, with 60 genera and 200 species [1], being grouped into 11 subfamilies: Macrotinae, Micronycterinae, Desmodontinae, Lonchorhininae, Phyllostominae, Glossophaginae, Lonchophyllinae, Carolliinae, Glyphonycterinae, Rhinophyllinae, and Stenodermatinae [2]. This family shows huge morphological, molecular and cytogenetic variation, both between and within species, in addition to much controversy about its taxonomy [2–5].

Traditionally, the subfamily Carolliinae consists of genera *Carollia* (10 spp.) and *Rhinophylla* (3 spp.) [3, 4, 6, 7]. However, in the classification proposed by Baker et al. [2] these two genera are not closely related. The subfamily Carolliinae (composed by the genus *Carollia*) was included as sister group to Glyphonycterinae, a new subfamily that includes *Glyphonycteris* and *Trinycteris* that were previously subgenera within *Micronycteris* [8–10], and later were raised to the genus level [3, 11]. *Rhinophylla* was included in its own subfamily (Rhinophyllinae) that was seen as closely related to the subfamily Stenodermatinae.

In the Baker et al. [2] classification, the group of Carolliinae + Glyphonycterinae + Rhinophyllinae + Stenodermatinae was designated Nullicauda, ​​an unranked taxon, initially proposed by Wetterer et al. [3] for the group consisting of subfamilies Carolliinae and Stenodermatinae.

Cytogenetic studies in species of the Nullicauda group show extensive interspecific and intraspecific chromosome variation [12]. To further investigate the controversial phylogenetic hypotheses based on morphological and molecular data in Nullicauda, ​​we analyzed chromosome evolution in this group through multidirectional chromosome painting using whole chromosome paint probes from two phyllostomid bats, *Phyllostomus hastatus* (PHA) and *Carollia brevicauda* (CBR) [13], in the species *Rhinophylla pumilio*, *Rhinophylla* aff. *fischerae*, *Trinycteris nicefori* and *Glossophaga soricina*. The results obtained here were compared with those previously published with the same probes in other species, in order to construct a phylogeny based on chromosomal homology data. We also integrated the comparative chromosome maps obtained with these probes with maps generated using human probes in *Glossophaga soricina* [14, 15].

## Methods

### Specimens examined

Chromosome banding and painting were used to analyze the karyotypes of bats from three phyllostomid subfamilies, Glossophaginae, Glyphonycterinae and Rhinophyllinae (Table 1), with the last two being members of the Nullicauda group. The specimens were captured with mist nets. Chromosome preparations and tissue biopsies were sent to the Cytogenetic Laboratory of the Federal University of Pará. Specimens were deposited in the Mastozoology collection of the Emilio Goeldi Museum and Zoology Museum of the Western Pará University.

**Table 1:** Species and karyotypes analyzed in the present work and data from literature used on mapping comparison

Scientific Names and Abbreviations	Subfamilies	2 N	FN	Locality	Number and sex	Cross-species FISH
*Rhinophylla aff. fischerae*^*a*^*,* RFI	Rhinophyllinae	38	68	Juruti (2°08′40”S; 56°05′28”W) and Santarém (2°26″54”S; 54°42″07”W)	3 (1 M, 2F)	This study
*Rhinophylla pumilio,* RPU	Rhinophyllinae	34	62	Santa Barbara (1°13′31”S; 48°17″51”W)	3 (2 M, 1F)	This study
*Glossophaga soricina,* GSO	Glossophaginae	32	60	Belém (1°28′05”S; 48°26′35”W)	1 (1F)	This study
*Trinycteris nicefori,* TNI	Glyphonycterinae	28	52	Oriximiná (1°45′40”S; 55°51′52”W) and Santarém (2°26″54”S; 54°42″07”W)	3 (1 M, 2F)	This study
*Phyllostomus hastatus,* PHA	Phyllostominae	32	60			Pieczarka et al. 2005
*Carollia brevicauda,* CBR	Carolliinae	20/21	36			Pieczarka et al. 2005
*Artibeus obscurus,* AOB	Stenodermatinae	30/31	56			Pieczarka et al. 2013
*Uroderma magnirostrum,* UMA	Stenodermatinae	36	62			Pieczarka et al. 2013
*Uroderma bilobatum,* UBI	Stenodermatinae	42	50			Pieczarka et al. 2013
*Chiroderma villosum*, CVI	Stenodermatinae^b^	26	48			Gomes et al. 2016
*Mesophylla macconnelli*, MMA	Stenodermatinae^b^	21/22	18			Gomes et al. 2016
*Vampyressa thyone*, VTH	Stenodermatinae^b^	23/24	20			Gomes et al. 2016
*Vampyriscus bidens*, VBI	Stenodermatinae^b^	26	48			Gomes et al. 2016
*Vampyriscus brocki*, VBR	Stenodermatinae^b^	24	44			Gomes et al. 2016

^a^The species here is called *aff*. (= *affinis*) because Gomes et al. (2010) demonstrated that, despite morphologically similar to *Rhinophylla fischerae*, its karyotype diverges clearly from the one described previously for that species by Baker and Bleier (1971) in a sample from Colombia. However, further studies are necessary to formally confirm that it is a different species. ^b^Subtribe Vampyressina. M = male; F = female

### Chromosome preparations, cell culture and chromosome banding

Metaphase chromosomes were obtained from direct bone marrow extraction [16], and through primary culture of fibroblasts [17]. G-banding patterns were obtained from incubation in enzymatic trypsin solution [18], and subsequent incubation in saline solution (0.5X SSC) and Wright dye staining diluted in phosphate buffer at a ratio of 1: 3 v / v. Staining time was 5 min. Karyotypes were organized following the pattern in the literature for each species.

### Fluorescence in situ hybridization (FISH)

Multidirectional chromosome painting was performed using whole chromosome probes from CBR and PHA (Phyllostomidae), generated by flow cytometry [13]. The probes were amplified and labeled by DOP-PCR [19, 20] and hybridized following procedures previously described [13, 20]. Briefly, the slides were incubated in a pepsin enzyme solution, washed in 2× SSC and dehydrated in an alcohol series (70%, 90% and 100%). Subsequently the slides were oven aged at 65 °C for 1 hour, denatured for 1 min in formamide solution (70% formamide/ 2× SSC) and incubated in hybridization solution (14 μl of solution containing: 50% formamide, 2× SSC, 10% dextran sulfate, 5 μg of salmon sperm DNA, 2 μg mouse Cot-1 DNA and 1 μl of labelled PCR product) for 3 days. After post-hybridization stringency washing, biotin-labeled probes were detected with avidin-Cy3 or avidin-FITC (1 μg/ml; Amersham). For dual color-FISH we used both biotin-labeled and direct-labeled probes. Digital images were captured using Axiovision 3.0 software via an Axiocam mRM CCD camera, coupled to a Zeiss Axioplan 2 microscope or with the Nis-Elements software on a Nikon H550S microscope. Chromosomes were identified by chromosomal morphology and by staining patterns with the fluorochrome DAPI (4 ‘, 6-diamidino-2-phenylindole).

### Literature data

The results obtained here were compared with those using PHA and CBR probes [13] previously published for *Chiroderma villosum*, *Mesophylla macconnelli*, *Vampyressa thyone*, *Vampyriscus bidens* and *Vampyriscus brocki* from subtribe Vampyressina [21]; *Artibeus obscurus*, *Uroderma bilobatum* and *Uroderma magnirostrum* from the subfamily Stenodermatinae [22], thus allowing phylogenetic analysis (see below) of the Nullicauda group. Finally, the mapping data performed here from *Glossophaga soricina* (GSO) were compared with those of [14, 15], allowing the establishment of chromosomal correspondence between PHA and CBR bats and human.

### Phylogenetic analysis using chromosomal characters

A binary matrix was constructed showing the presence and absence of discrete characters through chromosome homologies among the species analyzed in this paper and those from literature (see above) and is available as a Additional file 1. The results generated were used in a parsimony cladistic analysis. We used the PHA species as outgroup because 1) it is the most basal among the species analyzed here; 2) its karyotype is similar to *Macrotus californicus* (MCA), a species of the most basal genus of Phyllostomidae according to Baker et al. [2]; and 3) its karyotype is similar to the ancestral karyotype [22]. The analysis of Maximum Parsimony was made using PAUP 4.0b10 (Phylogenetic Analysis Using Parsimony) [23]. A heuristic search to find the most parsimonious tree(s) was performed using Tree Bisection Reconnection (TBR) branch-swapping; the posterior bootstrap probability was obtained with one thousand replicates.

## Results

### *Rhinophylla pumilio*

**Fig. 1:**
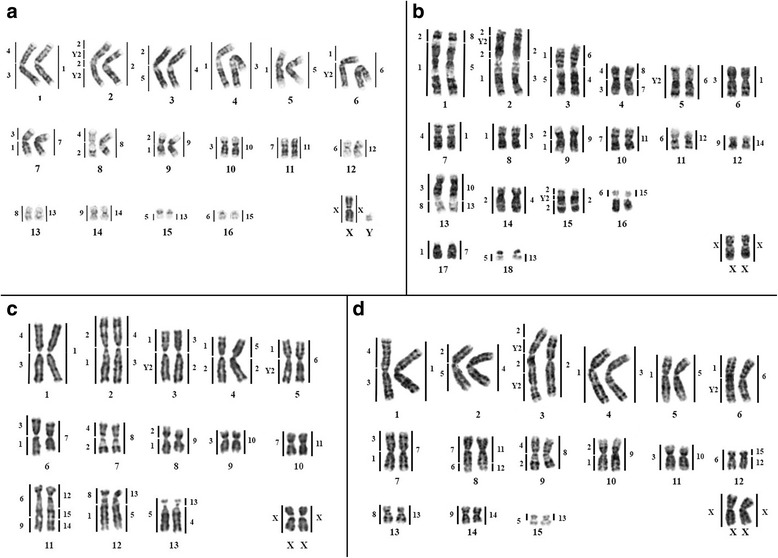
G-banded karyotype showing the mapping from CBR (left) and PHA (right) probes. **a** *Rhinophylla pumilio*. **b** *Rhinophylla* aff. *fischerae*. **c** *Trinycteris nicefori*. **d** *Glossophaga soricina*

*Rhinophylla pumilio* (RPU) shows a karyotype with 2n = 34, and FN = 62 (Fig. 1a), as well as G-, C-banding and Ag-NOR staining, FISH with telomeric and ribosomal DNA probes (not shown) similar to those from literature [24].

Hybridization of PHA whole chromosome probes onto the genome of RPU revealed 17 homologous segments (Fig. 1a). Fifteen PHA paints (PHA 1, 2, 3, 4, 5, 6, 7, 8, 9, 10, 11, 12, 14, 15 and X) are fully preserved in RPU 1, 2, 4, 3, 5, 6, 7, 8, 9, 10, 11, 12, 14, 16 and X chromosomes, respectively. Only PHA13 showed two hybridization signals, in RPU 13 and RPU 15q.

**Fig. 2:**
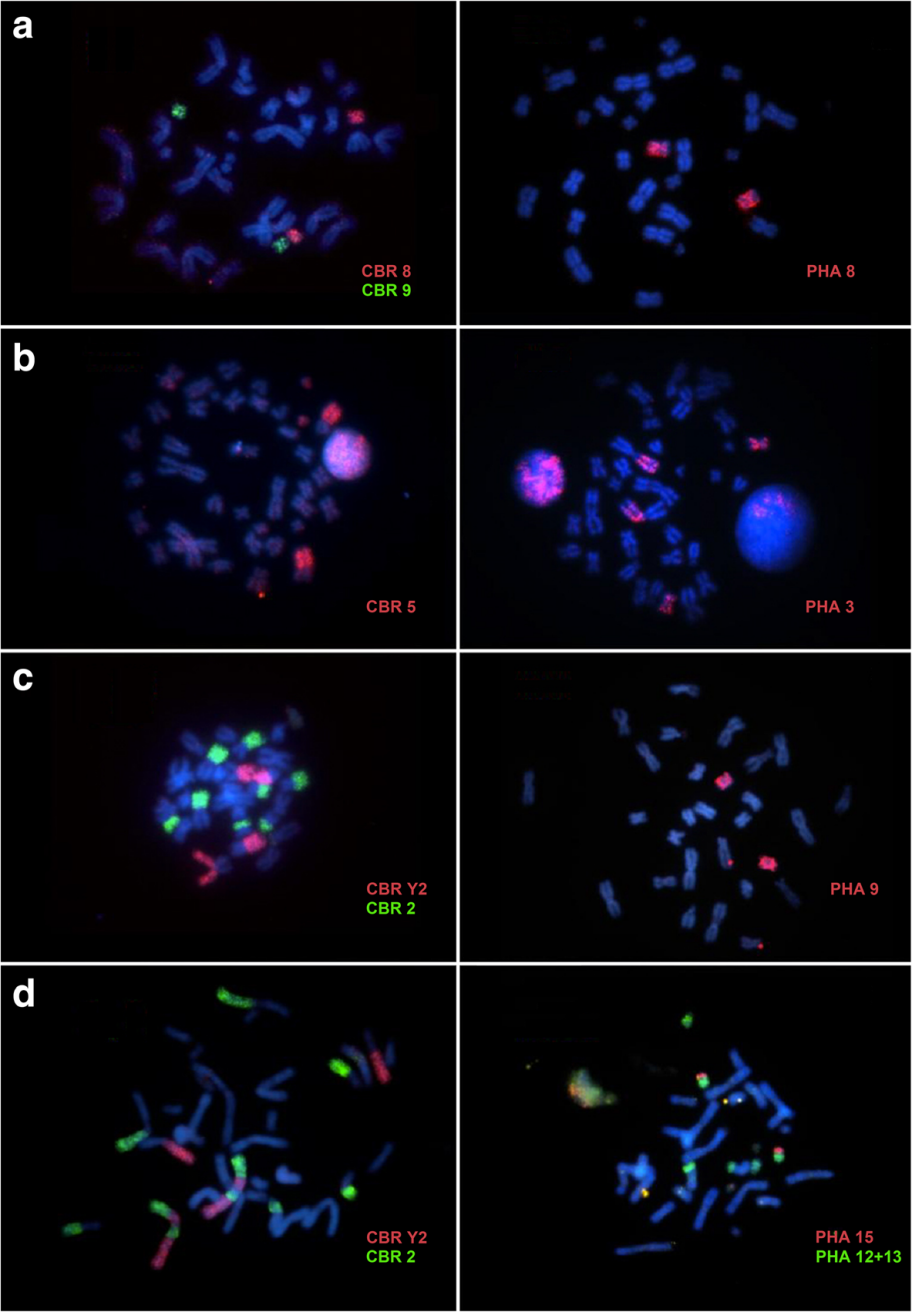
Examples of chromosome painting with probes from CBR (left) and PHA (right). The red probes were detected with Cy3 and the green with FITC. DAPI was used as counter staining. **a** RPU. **b** RFI. **c** TNI. **d** GSO

Hybridization of CBR whole chromosome probes onto the genome of RPU revealed 26 homologous segments (Fig. 1a). Eleven chromosome pairs of RPU hybridized with only one probe of CBR: RPU 4 (CBR 1), RPU 5 (CBR 1), RPU 10 (CBR 3), RPU 15q (CBR 5), RPU 12 (CBR 6), RPU 11 (CBR 7), RPU 13 (CBR 8), RPU 14 (CBR 9), RPU 16 (CBR 6), RPU X (CBR X) and RPU Y (CBR Y1). CBR probes 7, 8, 9 and X were fully conserved in RPU. Six chromosome pairs of RPU hybridized with two different probes of CBR: RPU 1 (CBR 3/4), RPU 3 (CBR 2/5), RPU 6 (CBR 1/Y2), RPU 7 (CBR 3/1), RPU 8 (CBR 4/2) and RPU 9 (CBR 2/1). One chromosome pair of RPU (RPU 2) hybridized with two probes of CBR, but with four signals: CBR 2/Y2/2/Y2. Examples of painting with CBR and PHA probes can be seen in Fig. 2a.

### Rhynophylla aff. fischerae

*Rhinophylla* aff. *fischerae* (RFI) has a karyotype with 2n = 38 and FN = 68 (Fig. 1b). The results on G-, C-banding and Ag-NOR staining, FISH with telomeric and ribosomal DNA probes (not shown) are similar to those from literature [25].

Chromosome painting with whole chromosome probes of PHA revealed 24 homologous segments in RFI (Fig. 1b). Fourteen chromosome pairs of RFI hybridized with only one probe of PHA each: RFI 5 (PHA 6), RFI 6 (PHA 1), RFI 7 (PHA 1), RFI 8 (PHA 3), RFI 9 (PHA 9), RFI 10 (PHA 11), RFI 11 (PHA 12), RFI 12 (PHA 14), RFI 14 (PHA 4), RFI 15 (PHA 2), RFI 16 (PHA 15), RFI 17 (PHA 7), RFI 18 (PHA 13) and RFI X (PHA X). Five chromosome pairs of RFI hybridized with two probes of PHA: RFI 1 (PHA 8/5); RFI 2 (PHA 2/3); RFI 3 (PHA 6/4); RFI 4 (PHA 8/7); RFI 13 (PHA 10/13).

The RFI genome revealed 29 hybridization signals with whole chromosome probes of CBR (Fig. 1b). Eleven chromosome pairs of RFI hybridized with only one probe of CBR each: RFI 5 (CBR Y2), RFI 6 (CBR 3), RFI 7 (CBR 4), RFI 8 (CBR 1), RFI 10 (CBR 7), RFI 11 (CBR 6), RFI 12 (CBR 9), RFI 14 (CBR 2), RFI 16 (CBR 6), RFI 17 (CBR 1), RFI 18 (CBR 5) and RFI X (CBR X). Five chromosome pairs of RFI hybridized with two probes of CBR: RFI 1 (CBR 2/1), RFI 3 (CBR 1/5); RFI 4 (CBR 4/3), RFI 9 (CBR 2/1) and RFI 13 (CBR 3/8). The chromosome pairs RFI 2 and RFI 15 had four (CBR 2/Y2/2/1) and three (CBR 2/Y2/2) hybridization signals, respectively. Pair RFI 16 hybridized only in its short arm (CBR 6), while the long arm was fully heterochromatic. Examples of painting with CBR and PHA probes can be seen in Fig. 2b.

### Trinycteris nicefori

*Trinycteris nicefori* (TNI) has a karyotype with 2n = 28 and FN = 52 (Fig. 1c), a similar result to those from literature [26]. The chromosome complement consists of 10 meta/submetacentric and three subtelocentric chromosomes. The X chromosome is submetacentric and the Y is acrocentric. The sequential G-, C-banding pattern showed constitutive heterochromatin blocks in the pericentromeric region of all chromosomes and faint blocks in the distal portion of the short arm of pairs 4 and 6. Staining with silver nitrate showed the NOR in the proximal region of the short arm of pair 6. Fluorescence in situ hybridization with telomeric probes showed signs only at chromosomal tips and 18S rDNA probes confirmed the silver nitrate staining. These data were used to confirm the karyotype analysis but are not shown here.

Chromosome painting with whole chromosome probes of CBR and PHA onto the genome of TNI revealed 24 and 21 conserved homologous segments, respectively (Fig. 1c). Eight PHA paints (PHA 1, 6, 7, 8, 9, 10, 11 and X) are fully preserved in TNI 1, 5, 6, 7, 8, 9, 10 and X chromosomes, respectively. Five TNI hybridized with two different probes of PHA: TNI 2 (PHA 4/3), TNI 3 (PHA 3/2), TNI 4 (PHA 5/2), TNI 12 (PHA 13/5) and TNI 13 (PHA 13/4). Only TNI 11 hybridized with 3 PHA probes (PHA 12/15/14).

Hybridizations with CBR probes showed that four TNI pairs hybridized with a single CBR probe each: TNI 9 (CBR 3), TNI 10 (CBR 7), TNI 13 (CBR 5) and TNI X (CBR X). The remaining pairs hybridized with two CBR probes each: TNI 1 (CBR 4/3), TNI 2 (CBR 2/1), TNI 3 (CBR 1/Y2), TNI 4 (CBR 1/2), TNI 5 (CBR 1/Y2), TNI 6 (CBR 3/1), TNI 7 (CBR 4/2), TNI 8 (CBR 2/1), TNI 11 (CBR 6/9) and TNI 12 (CBR 8/1). Examples of painting with CBR and PHA probes can be seen in Fig. 2c.

### *Glossophaga soricina*

*Glossophaga soricina* (GSO) has a karyotype with 2n = 32 and FN = 60 (Fig. 1d). These data, as well as those of classical cytogenetics in this species, are similar to those from the literature [14, 15, 27]. The karyotype was organized following Sotero-Caio et al. [15], with the aim of comparing the PHA and CBR probes with human probes (see below).

Chromosome painting with whole chromosome probes of CBR and PHA onto the genome of GSO revealed 18 and 26 conserved homologous segments (Fig. 1d). Fourteen chromosome pairs of GSO hybridized with only one probe of PHA each: GSO 1 (PHA 1), GSO 2 (PHA 4), GSO 3 (PHA 2), GSO 4 (PHA 3), GSO 5 (PHA 5), GSO 6 (PHA 6), GSO 7 (PHA 7), GSO 9 (PHA 8), GSO 10 (PHA 9), GSO 11 (PHA 10), GSO 13 (PHA 13), GSO 14 (PHA 14), GSO 15 (PHA 13) and GSO X (PHA X). Two chromosome pairs of GSO hybridized with two probes of PHA: GSO 8 (PHA 11/12) and GSO 12 (PHA 15/12).

Eight chromosome pairs of GSO hybridized with only one probe of CBR each: GSO 4 (CBR 1), GSO 5 (CBR 1), GSO 11 (CBR 3), GSO 12 (CBR 6), GSO 13 (CBR 8), GSO 14 (CBR 9), GSO 15 (CBR 5) and GSO X (CBR X). Seven chromosome pairs of GSO hybridized with two probes of CBR: GSO 1 (CBR 3/4), GSO 2 (CBR 2/5), GSO 6 (CBR 1/Y2), GSO 7 (CBR 3/1), GSO 8 (CBR 7/6), GSO 9 (CBR 4/2) and GSO 10 (CBR 2/1). Pair GSO 3 hybridized with two probes of CBR, but with four signals: CBR 2/Y2/2/Y2. Examples of painting with CBR and PHA probes can be seen in Fig. 2d.

### Correspondence among PHA, CBR and human whole chromosome probes

**Fig. 3:**
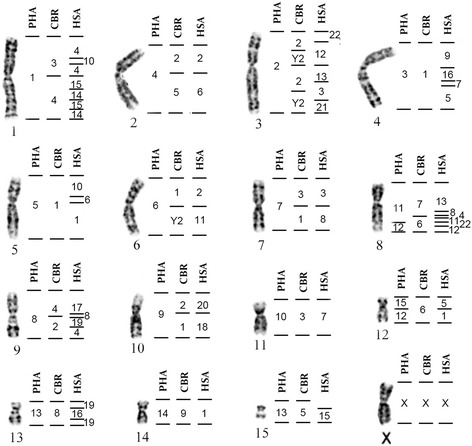
Homeology of the syntenic groups of PHA and CBR (present study) with HSA (Sotero-Caio et al., 2013) using GSO chromosomes as reference

Once we mapped the GSO genome with the PHA and CBR probes, we were able to determine the correspondence of these probes with human (HSA) chromosomes, since previous works [14, 15] performed HSA mapping in GSO (Fig. 3).

### Phylogenetic analyses using chromosomes as characters

**Fig. 4:**
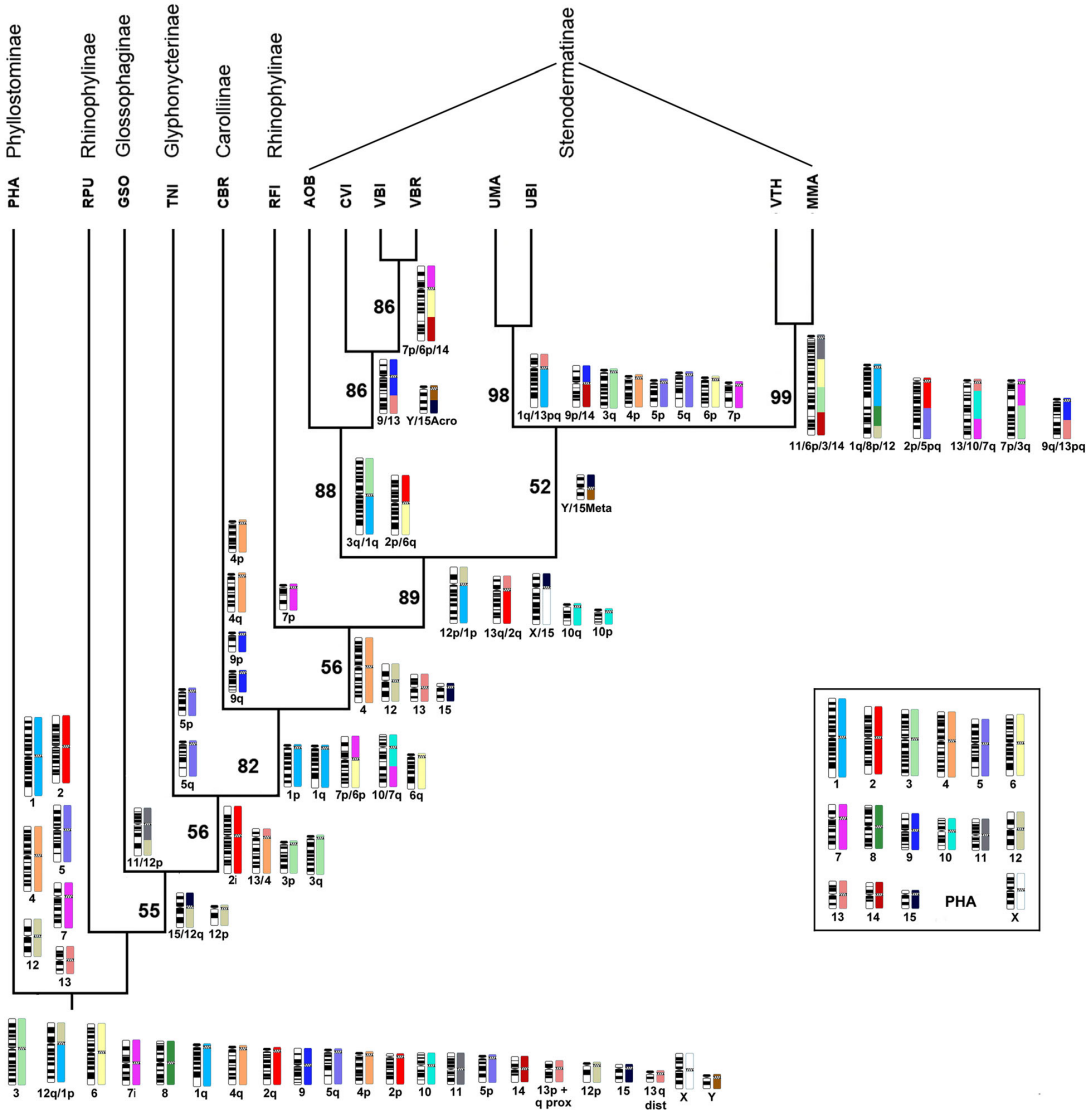
Maximum parsimony tree obtained with PAUP software, using chromosome characters resulted from comparative mapping of the species here studied. Abbreviations are described in Table 1. Other symbols: “p” = short arm; “q” = long arm; “meta” = metacentric chromosome; “acro” = acrocentric chromosome; “/” = syntenic groups physically linked; “i” = inversion; “p + q” = part of short arm linked with part of the long arm. On the top are the subfamilies. Bold numbers (over the branches) are the bootstrap values for one thousand replicates. Ideograms: 1) into the box are the chromosomes of *Phyllostomus hastatus*, used as probes; each chromosome is represented by G-banding and a specific color. The numbering on all the figure are related to this species karyotype, the outgroup. 2) Below the tree is the proposed ancestral karyotype of Phyllostomidae [22]. 3) Below each branch are represented the chromosomes found on each species or group of species karyotypes which resulted from rearrangements and are relevant to the present analysis

Based on chromosome homologies identified by multidirectional chromosome painting, we analyzed the genera here mapped, together with the previously mapped PHA, CBR, *Uroderma magnirostrum* (UMA, 2n = 36, FN = 62), *Uroderma bilobatum* (UBI, 2n = 42, FN = 50), *Artibeus obscurus* (AOB, 2n = 30/31, FN = 56), and the Vampyressina *Chiroderma villosum* (CVI, 2n = 26, FN = 48), *Mesophylla macconnelli* (MMA, 2n = 21/22, FN = 18), *Vampyressa thyone* (VTH, 2n = 23/24 FN = 20), *Vampyriscus bidens* (VBI, 2n = 26, FN = 48) and *Vampyriscus brocki* (VBR, 2n = 24, FN = 44). We used a total of 93 discrete chromosomal characters to build a matrix of their presence or absence (Additional file 1: Table S1). The Maximum Parsimony analysis (MP) resulted in nine equally parsimonious trees (Tree length = 124, Consistence index = 0.75, retention index = 0.7578, Homoplasy index = 0.25). The main branch leads to all analyzed species except the outgroup (Fig. 4). After the split from PHA, the next branch leads to RPU (Rhinophyllinae), then GSO (Glossophaginae), TNI (Glyphonycterinae), CBR (Carolliinae), RFI (Rhinophyllinae) and the Stenodermatinae. In the last subfamily the phylogenetic relationships included Vampyressina, previously analyzed [21]. The present study confirmed the internal relationships of Stenodermatinae presented in that paper. RPU and RFI belong to the same subfamily but are in different branches because of the lack of phylogenetic signal in RPU (see Discussion).

### Mapping of chromosome changes in the phylogeny obtained

Comparing the data matrix with the phylogeny of Fig. 4 it was possible to list the chromosomal rearrangements that served as chromosome signatures for each branch.

## Discussion

Whole chromosomes probes from CBR and PHA, previously described [13], were hybridized onto the genomes of RPU, RFI, TNI and GSO in order to establish chromosomal homologies between these four species. Our results were integrated with those present in the literature for both these probes and also for human probes.

### Intra-specific and intrageneric chromosome variation in Rhinophylla

Multidirectional chromosome painting in RFI compared to RPU show many rearrangements between the two species. When comparing these results with Colombian RFI [28], the karyotype of Colombian RFI is similar to the RPU analyzed here, which emphasizes the differences between RFI of the two regions (Colombia and Brazil). These differences support the hypothesis that postulate the RFI from Brazil as a new species [25], *Rhinophylla* aff. *fischerae*, based on its reproductive isolation from the RFI of Colombia (due to multiple chromosomal rearrangements).

### Karyotypes and phylogeny

When comparing the nine equally parsimonious phylogenies obtained, we found that they were similar, with some differences due to polytomy. Considering the molecular phylogenies already published [1, 2] we opted for the phylogeny presented in Fig. 4. Analyzing the phylogeny, it is necessary to take into account that RPU, GSO and TNI show practically identical karyotypes to PHA, which in turn is similar to the MCA karyotype and to the ancestral karyotype of Phyllostomidae [22]. Thus the branching that places RPU near PHA is artificial and reflects the absence of phylogenetically informative signal in the RPU karyotype. This occurs more or less similarly with GSO and TNI, although some phylogenetic signal is shared with CBR, placing them in an intermediate position (see below). Thus, except for the RPU position, the phylogeny is similar to [1]. The recurrence of the karyotype similar to the hypothetical ancestral karyotype of Phyllostomidae throughout this phylogeny confirms its origin before the diversification of most subfamilies, implying that each subfamily developed its karyotypes independently from the ancestral karyotype. This would occur, for example, if the diversification of the branches that formed the subfamilies occurred in a short period of time. Evidence that this actually occurred was obtained by concatenating mitochondrial genomes with nuclear genes [29]. This fact explains the considerable difficulty in constructing Phyllostomidae phylogenies from chromosome data (even with precise tools such as chromosome painting) due to the few synapomorphies between subfamilies, which can be seen in the data matrix (Additional file 1).

### The ancestral karyotype of Phyllostomidae and chromosome signatures

Placing the chromosomal rearrangements in the phylogeny (Fig. 4) shows that almost all nodes are characterized by chromosomal rearrangements. We highlight the rearrangement PHA 7q10, which is present from CBR to MMA, uniting the subfamilies Carolliinae, Rhinophyllinae and Stenodermatinae. PHA 12/15, PHA 13/4 and the inversion in PHA 2p2q groups TNI and CBR species. Thus, although bootstrap values are eventually low, the occurrence of these rearrangements generates considerable confidence in this phylogeny.

### Shared rearrangements with other families

The correspondence among the human whole chromosome probes with PHA and CBR (Fig. 3) allows the comparison of the Phyllostomidae karyotypes with representatives of other families where the human probes were mapped. A comparison of the human syntenic associations present in the karyotypes of several families was previously made [30]. For Phyllostomidae they used the mapping of GSO [14]. Yangochiroptera (superfamilies Emballonuroidea, Noctilionoidea and Vespertilionoidea) the association HSA 5/7/16 was shared. With the exception of Emballonuridae, the other families shared HSA 4/10/14/15/14/15, HSA 7a/7b and HSA 18/20. HSA 13/8/4 is an association even older, shared between Yangochiroptera and Megadermatidae (Yinpterochiroptera). Thus, all these associations already existed before the appearance of Phyllostomidae and therefore were part of the ancestral karyotype of the family. HSA 5/7/16 corresponds to PHA 3q; HSA 4/10/14/15/14/15 to PHA 1; HSA 7a/7b to PHA 10; HSA 18/20 to PHA 9 and HSA 13/8/4 to PHA 11 (Fig. 3). The PHA chromosomes mentioned above are conserved in most of the analyzed species of Phyllostomidae and are also present in the family ancestral karyotype [22], with the exception of PHA 1, which is separated as PHA 1p (HSA 4 / 10) and PHA 1q (HSA 14/15/14/15). The same occurs in MCA [15], where HSA 4/10 corresponds to MCA 5 and HSA 14/15/14/15 to MCA 10. One point raised is that Robertsonian rearrangements are prone to homoplasy [30]. Thus, although PHA, GSO, RPU and TNI share the association found in Molossidae, Phyllostomidae and Vespertilionidae, the question remains whether the ancestral karyotype of Phyllostomidae had the chromosome arms corresponding to PHA 1 associated or separated.

Finally, the association HSA 4/10 is reshuffled via an inversion, which resulted in the HSA 4/10/4 arrangement in all species of Phyllostomidae so far studied. However, this inversion is absent from the other families of Chiroptera, representing a potential chromosomal signature exclusive for Phyllostomidae.

## Conclusions

In conclusion, although the phylogenetic power of chromosome painting data has somehow been affected by the rapid radiation of subfamily lineages, the data here presented show the chromosome signatures that support the validity of the Nullicauda by demonstrating phylogenetic associations between Rhinophyllinae-Stenodermatinae and Carolliinae-Glyphonycterinae.

## Additional file

Additional file 1: **Table S1.** Basic data matrix on chromosome rearrangements. The numbers of chromosomes are the ones from PHA, the outgroup. The meaning of the abbreviations can be found at Table 1. Other symbols: “/” = syntenic groups physically linked; “i” = inversion; “p” = short arm; “q” = long arm; “p + q” = part of short arm linked with part of the long arm; “-”= part of the arm is missing. (DOCX 158 kb)

## Abbreviations

2n: Diploid number
Ag-NOR: Silver nitrate staining
AOB: *Artibeus obscurus*
CBR: *Carollia brevicauda*
CVI: *Chiroderma villosum*
DAPI: 4′, 6-diamidino-2-phenylindole
DOP-PCR: Degenerate Oligonucleotide-Primed-Polymerase Chain Reaction
FISH: *Fluorescence* In Situ *Hybridization*
FN: Fundamental Number
GSO: *Glossophaga soricina*
HSA: *Homo sapiens*
MCA: *Macrotus californicus*
MMA: *Mesophylla macconnelli*
MP: Maximum parsimony
NOR: Nucleolar Organizer Region
PAUP: Phylogenetic Analysis Using Parsimony
PHA: *Phyllostomus hastatus*
RFI: *Rhinophylla* aff. *fischerae*
RPU: *Rhinophylla pumilio*
SSC: Saline Sodium Citrate
TBR: Tree Bisection Reconnection
TNI: *Trinycteris nicefori*
UBI: *Uroderma bilobatum*
UMA: *Uroderma magnirostrum*
VBI: *Vampyriscus bidens*
VBR: *Vampyriscus brocki*
VTH: *Vampyressa thyone*
